# Successful Treatment of Myeloid Sarcoma in an Elderly Patient with Myelodysplastic Syndrome with Reduced-Dose Azacitidine

**DOI:** 10.1155/2021/6640597

**Published:** 2021-04-21

**Authors:** Kazuya Sato, Nodoka Tsukada, Junki Inamura, Shigetsuna Komatsu, Keisuke Sato, Masayo Yamamoto, Motohiro Shindo, Kentaro Moriichi, Yusuke Mizukami, Mikihiro Fujiya, Yoshihiro Torimoto, Toshikatsu Okumura

**Affiliations:** ^1^Department of Hematology/Oncology, Asahikawa Kosei General Hospital, 1-24, Asahikawa 078-8211, Japan; ^2^Department of Dermatology, Asahikawa Kosei General Hospital, 1-24, Asahikawa 078-8211, Japan; ^3^Department of Clinical Laboratory, Asahikawa Kosei General Hospital, 1-24, Asahikawa 078-8211, Japan; ^4^Division of Gastroenterology and Hematology/Oncology, Department of Medicine, Asahikawa Medical University, Asahikawa, Hokkaido, Japan

## Abstract

Myeloid sarcoma (MS), which involves extramedullary lesions, is classified as a unique subtype of acute myeloid leukemia (AML). At present, no standard treatments for MS have been established. The patient was an 89-year-old man with myelodysplastic syndrome-excess blast-2 (MDS-EB-2) with a 2-year history of intermittent treatment with azacitidine (AZA) during a 4-year history of MDS. He developed painful cutaneous tumors 8 months after the second discontinuation of AZA. They were refractory for antibiotics and topical tacrolimus hydrate. A tumor biopsy was performed, and the histological findings of the tumor lesion showed a proliferation of tumor cells that were positive for myeloperoxidase and CD68 and negative for CD4 and CD123. The patient was diagnosed with MDS-associated MS. MDS-EB-2 quickly progressed to AML with the appearance of peripheral blood blasts and 25% bone marrow blasts. Monotherapy with reduced-dose AZA (37.5 mg/m^2^ for 7 days, every 4–6 weeks) was restarted, and the MS quickly disappeared. The patient's MS was successfully treated with 16 cycles of AZA treatment over a 22-month period. There have been 10 reported cases in which MS was successfully treated with AZA. Among the 10 cases, the patient in the present case was the oldest. Treatment with reduced-dose AZA should be considered as a therapeutic option for MS in elderly patients with MDS, especially patients who are ineligible for intensive chemotherapy.

## 1. Introduction

Myeloid sarcoma (MS), which involves extramedullary lesions at sites such as the skin, lymph nodes, gastrointestinal tract, bone, soft tissue, and testis, is classified as a unique subtype of acute myeloid leukemia (AML) [1–3]. MS may precede or coincide with AML or represent acute blastic transformation of myelodysplastic syndrome (MDS), myeloproliferative neoplasms, or chronic myelogenous leukemia [1, 2, 4]. No standard treatment for MS has been established; thus, intensive chemotherapy consisting of anthracycline and cytarabine is generally selected as the initial therapy, according to chemotherapy for AML. In addition, allogeneic hematopoietic stem-cell transplantation (allo-HSCT) or radiation therapy may also be considered [2, 4]. However, elderly patients are generally not eligible for intensive chemotherapy and allo-HSCT; thus, further strategies are required, especially for elderly patients with MS.

A hypomethylating agent, azacitidine (AZA), which improved overall survival in comparison to conventional care treatment in higher-risk MDS patients who were not eligible for allo-HSCT [5], is widely used in clinical practice for the treatment of patients with MDS, as well as a proportion of patients with AML [6, 7]. However, there are few reports of patients with MS who were successfully treated with AZA [8–13].

We herein report a case of MS in an elderly patient with MDS, which was successfully treated with 16 cycles of AZA treatment over a 22-month period. This is a rare case of MS that was successfully treated with AZA monotherapy.

## 2. Case Presentation

**Figure 1:**
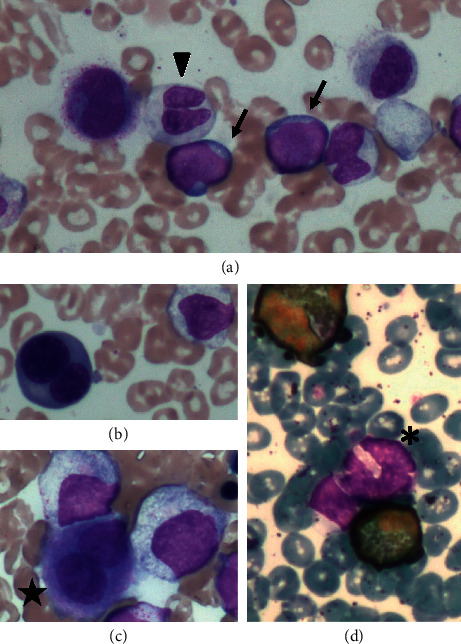
Microscopic findings of the bone marrow smears. Bone marrow aspirate smears (May–Giemsa staining; magnification: ×400) showed (a) myeloblasts (arrows) and a hyposegmented mature neutrophil (arrowhead), (b) a binucleated erythroblast, and (c) a micromegakaryocyte (indicated with a star). (d) Myeloperoxidase staining (magnification: ×400) of the bone marrow cells showed a hypogranular neutrophil (indicated with an asterisk).

An 85-year-old man with pancytopenia was diagnosed with MDS with multilineage dysplasia (MDS-MLD) based on bone marrow aspiration (BMA), which revealed 2.6% myeloblasts with trilineage dysplasia and normal karyotype. Based on these findings, the case was categorized as intermediate-1 or low risk according to the International Prognostic Scoring System (IPSS) [14] or Revised IPSS (IPSS-R) [15], respectively. At this point, the patient had been treated with vitamin K2 (menatetrenone) and active vitamin D3 (calcitriol) because combination therapy of these two drugs was reported to be effective against low-risk MDS [16]. At 87 years of age, the patient undertook BMA again because his pancytopenia had progressed, and he was diagnosed with MDS with excess blasts-2 (MDS-EB-2) based on the BMA findings, which revealed 11% myeloblasts and multilineage dysplastic changes of bone marrow cells (Figures 1(a)–1(d)), with a normal karyotype. The disease status was categorized as intermediate-2 risk according to the IPSS and high risk according to the IPSS-R; thus, AZA treatment was initiated on hospitalization at the standard dose of 75 mg/m^2^/day, subcutaneously, for 7 days every month. The patient achieved a hematological improvement (HI) with manageable adverse events (AEs), including constipation and general malaise after the first cycle of AZA. The second cycle was therefore administered in an outpatient setting. However, the treatment was discontinued due to septic shock after the second cycle of AZA. The patient became dependent on red blood cell (RBC) transfusions due to a clinical progression of MDS because one year after the discontinuation of AZA (at 88 years of age), we did not perform any histological examinations, such as BMA. Therefore, additional 3 cycles of AZA were administered in an outpatient setting with a reduced dose of 37.5 mg/m^2^ (50% dose reduction) daily. The dose of AZA was decided according to the prescribing information for AZA [17] based on the nadir absolute neutrophil count <0.5 × 10^9^/L in the preceding (second) cycle. Treatment with AZA was discontinued again because the patient refused to continue the treatment due to the AEs, which included constipation and general malaise, as well as the inconvenience of going to the hospital.

**Figure 2:**
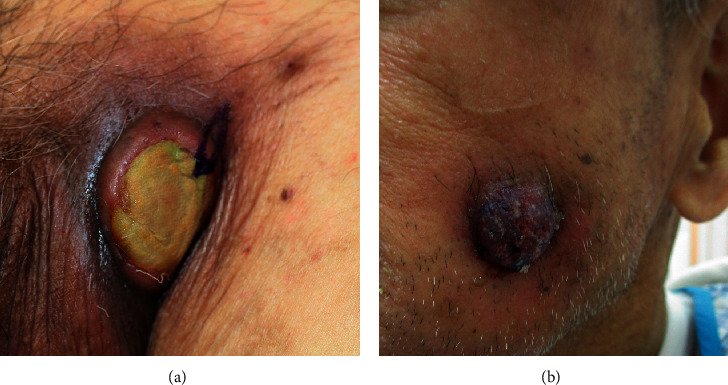
Cutaneous tumor lesions. (a) A painful cutaneous tumor, the top of which showed yellow-colored necrosis, which developed at the left groin 8 months after the second discontinuation of azacitidine. (b) Another cutaneous tumor, which appeared on the left cheek one month after the development of the first cutaneous lesion at the left groin.

**Figure 3:**
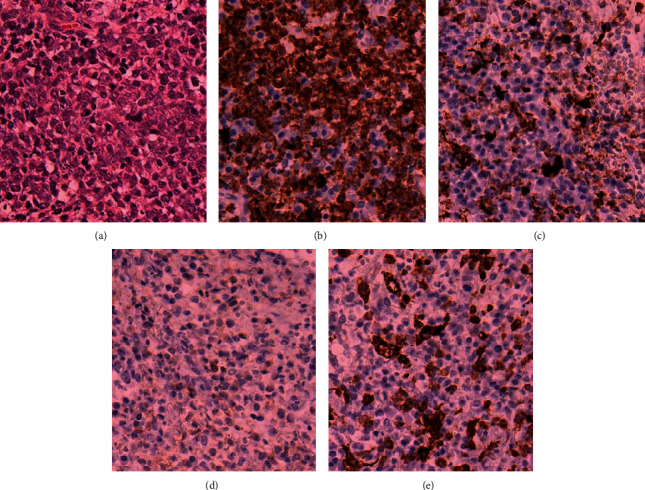
The histological findings of the tumor located in the left groin. A biopsy specimen showed the proliferation of tumor cells with a high nuclear/cytoplasmic ratio on hematoxylin and eosin staining (×20) (a). Immunohistochemical staining (×20) of tumor cells was positive for myeloperoxidase (b), partially positive for cluster of differentiation (CD) 68 (c), negative for CD4 (d), and negative for CD123 (e).

At eight months after the second discontinuation of AZA (at 89 years of age), a painful cutaneous tumor, the top of which showed yellow-colored necrosis (Figure 2(a)), developed at the left groin lesion. Laboratory tests revealed the following: white blood cell count, 2,900/*μ*L, with 63.6% neutrophils, 32.6% lymphocytes, 3.5% monocytes, 0.3% eosinophils, and no blast cells; hemoglobin, 6.6 g/dL; platelet count, 8.7 × 10^4^/*μ*L; and C-reactive protein, 0.57 mg/dL. No treatments, including oral and topical antibiotics, topical tacrolimus hydrate, and analgesics, had any effect on the tumor. A biopsy of the tumor was performed one month after its development. Histological examination revealed the proliferation of tumor cells with a high nuclear/cytoplasmic ratio (Figure 3(a)). Immunohistochemically, the neoplastic cells were positive for myeloperoxidase (Figure 3(b)), partially positive for cluster of differentiation (CD) 68 (Figure 3(c)) and CD33, but negative for CD3, CD4 (Figure 3(d)), CD20, CD34, CD56, CD117, and CD123 (Figure 3(e)). A diagnosis of MDS-associated MS was made. One month after the development of the first cutaneous lesion, another cutaneous tumor appeared on the left cheek (Figure 2(b)). BMA revealed a hypercellular marrow with 25% myeloblasts, which were positive for CD13, CD33, CD34, and HLA-DR; a G-band analysis revealed a normal karyotype. A diagnosis of AML secondary to MDS was made.

**Figure 4:**
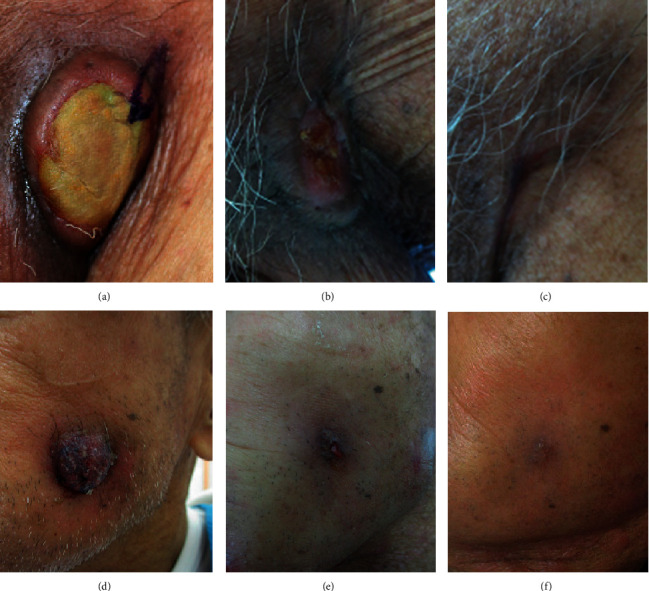
The response of cutaneous myeloid sarcoma (MS) to treatment with azacitidine. An MS located at the left groin before the first (6th in total) cycle of azacitidine retreatment (a). Before the second (7th in total) cycle (b). After the second cycle (c). An MS on the left cheek before the first cycle (d). Before the second cycle (e). After the second cycle (f).

Considering the patient's age, clinical conditions, and the previously experienced AEs, treatment with AZA was started again at a dose of 37.5 mg/m^2^/day, subcutaneously, for 7 days every 4–6 weeks (in total, this was the 6^th^ cycle of AZA treatment) during short-term hospitalization, which consisted of 9 days of inpatient treatment per cycle, in order to manage the acute-phase AEs caused by AZA. The cutaneous MS lesion located on the left groin (Figures 4(a)–4(c)) and that on the left cheek (Figures 4(d)–4(f)) promptly disappeared after the 6^th^ and 7^th^ cycles of AZA treatment. Although myeloblasts had appeared in peripheral blood after several cycles of retreatment with AZA, the treatment was continued because of persistence of the response on MS, and the pain caused by his lesions remained relieved, with tolerable AEs, by AZA. The patient had received 16 cycles (over a 22-month period) of AZA treatment with the same dose on repeated short-term hospitalization until he died of progression of AML. He eventually received 21 cycles of AZA treatment in total. No exacerbation of cutaneous MS had been confirmed during AZA treatment.

## 3. Discussion

We experienced a case of MS in an elderly man with MDS, which was successfully treated with AZA over a 22-month period. Although cytarabine-based intensive chemotherapy is generally selected as the initial therapy for MS (based on its efficacy) [2, 3], the patient in the present case was 89 years of age, and there were concerns about AEs. Radiation therapy may be a therapeutic option for MS; however, the patient had already developed multiple MS lesions; thus, it was not selected. For these reasons, reduced-dose AZA monotherapy was restarted again, and AZA-associated AEs were managed by short-term hospitalization. While the present patient was expected to have an approximately 2-year survival after starting AZA treatment, based on the result of the AZA-001 phase III trial for higher-risk MDS (median overall survival of 24.5 months) [5], he received 21 cycles of AZA treatment in total and eventually survived for 49 months despite developing MS.

To our knowledge, there are 10 reported cases in which MS including leukemia cutis (LC), a phenotype of extramedullary leukemia induced by the infiltration of leukemic cells into the epidermis, the dermis, or the subcutis [18], was successfully treated with AZA [8–13]. The clinical characteristics of the cases are shown in Table 1. Aside from two cases, most were elderly patients; our patient was the oldest. In all of these cases, AZA treatment was performed for MS that developed in a patient with preceding hematological malignancies (MDS, chronic myelomonocytic leukemia (CMML), monoblastic LC, or AML). AZA treatment was effective for MS originating at various sites; 7 cases (including our case) involved skin lesions. Four cases (cases 1 and 7–9) with MS were successfully treated with AZA in combination with sequential radiotherapy, multidrug chemotherapy, or biomodulatory drugs. One of the 4 cases (case 8) was treated with AZA as a maintenance therapy. Including the present case—in which AZA monotherapy was administered for remission induction and maintenance therapy—six cases (cases 2–6 and 10) with MS were successfully treated with AZA monotherapy, and five of them had skin leukemic lesions. MS resolved clinically, radiologically, or pathologically with AZA treatment in six cases (cases 3–5 and 8–10). One to 41 cycles of AZA therapy were performed for the 10 patients with MS; 6 of the patients eventually died of the progression of AML or other causes. The dose of AZA was reduced in 4 cases (cases 2, 4, 9, and 10) or modified in 1 case (case 8) among the 10 patients, and the present patient received the lowest dose of AZA (37.5 mg/m^2^ for 7 days) among the cases with repeated treatment for a long period. From these findings, AZA probably has some benefit for MS in a certain group of patients with myeloid malignancies.

Sporadic case studies [19–23] have reported that decitabine—another hypomethylating agent that is used for the treatment of MDS or for the treatment of elderly patients with AML who are not eligible for standard chemotherapy in Western countries—was effective for treating MS in patients with myeloid malignancies (e.g., AML or CMML). Some of those cases with MS involved the skin [19, 22], similar to our case. As shown in Table 1, successful treatment with AZA in MS patients with involvement of the skin or with LC has also been reported [9, 10, 12, 13]. Thus, hypomethylating agents may have some effects in certain patients with MS, including patients who develop cutaneous lesions.

In this case, the appearance of blasts in peripheral blood was observed after AZA treatment, which eventually led to the progression of leukemia, but the exacerbation of the skin lesions was not observed during the treatment. The first reason for this issue would be that a dose of sufficient intensity for the marrow or peripheral leukemic cells could not be maintained due to the dose reduction and the prolonged treatment interval (>4 weeks). Second, the activation of the immune system by AZA treatment may contribute to the antitumor effect on the skin MS lesions. It has been reported that AZA induces cytotoxic CD8 T cells for some tumor antigens, such as melanoma-associated antigens (MAGE antigens) and Wilm's tumor antigen 1 [24]. In addition, it has been reported that AZA and valproate upregulate MAGE antigens in AML cell lines [25]. Antigen-presenting cells, such as dendritic cells, which are necessary for the activation of antigen-specific cytotoxic T cells, are abundant in the skin. Thus, leukemic cells in the MS lesions on the skin might be controlled by not only the inhibition of DNA synthesis by AZA treatment but also by the antileukemic effect of the activation of cytotoxic T cells against the leukemia-specific antigens. Further accumulation of such cases is required to clarify this issue.

However, a case of LC has been reported in a patient with acute myelomonocytic leukemia on AZA treatment [26]. That case was considered refractory to the AZA treatment. The responsiveness of cutaneous lesions of MDS/AML to AZA treatment might differ by disease status or histology of underlying disease. Physicians should consider the possibility of such a case developing.

We did not have a good treatment plan for this patient other than repeating the treatment with AZA. Venetoclax, which is an inhibitor of the antiapoptotic BCL2 protein, is reported to be clinically beneficial in hypomethylating agent- (HMA-) resistant patients with MDS/AML in combination with an HMA [27]. While venetoclax is not currently available for patients with MDS/AML in our country, combination therapy of venetoclax and AZA will likely be a good treatment option for patients such as the present case.

## 4. Conclusion

We reported a case of MS that developed in an elderly patient with MDS, which was successfully treated with AZA monotherapy for a 22-month period, despite a reduction in the dose. Although further accumulation of such cases is needed, treatment with AZA should be considered as a potential treatment strategy when MS develops in patients with myeloid malignancies, such as MDS, especially elderly patients who are ineligible for intensive treatment. If an effect on MS is confirmed in such cases, AZA treatment should be continued as much as possible.

## Figures and Tables

**Table 1:** The clinical characteristics of cases of myeloid sarcoma that were successfully treated with azacitidine.

Case/reported year	Age/sex	Diagnosis at development of MS	Sites of origin	Treatment for MS	Cycles of AZA for MS	Maximum response of AZA for MS	Outcome (cause of death)	Reference
1 (2013)	62/M	CMML type-2	Right humerus	AZA (75 mg/m^2^ × 7 days) + RT (25 Gy)	6^*∗*^	Decreased in size	Alive	[8]
2 (2013)	34/M	Monoblastic leukemia cutis	Multiple skin nodules, groin mass	IDR + Ara C followed by allo-HSCT ⟶ CLAG followed by DLI and RT ⟶ a cycle of AZA (32 mg/m^2^ × 5 days) ^⟶^ 4 cycles of AZA (75 mg/m^2^ × 7 days) ⟶ RT	5	Decreased in size	Died (multiorgan failure)	[9]
3 (2013)	39/F	Relapse of AML-M2 after allo-HSCT	Gingiva	FLAG with GO ⟶ FLAG and DLI ⟶ AZA (75 mg/m^2^ × 7 days)	17^*∗*^	Clinical resolution	Alive	[9]
4 (2015)	72/M	MDS EB-2	Disseminated skin papules (LC)	36 cycles of AZA (75 mg/m^2^ × 5 days) for MDS ⟶ 41 cycles of the same dose of AZA after the onset of LC	41^*∗*^	Pathological resolution	Alive	[10]
5 (2015)	78/M	MDS MLD	Multiple skin lesions (LC)	AZA (75 mg/m^2^ × 7 days) ⟶ 6-MP	7	Clinical resolution	Died (N/A)	[10]
6 (2015)	76/M	CMML type-1	Multiple skin lesions (LC)	9 cycles of AZA (75 mg/m^2^ × 5 days) for CMML ⟶ 4 cycles of AZA (75 mg/m^2^ × 7 days) after the onset of LC	4	Regression of LC	Died (N/A)	[10]
7 (2016)	80/F	MDS ⟶ AML	Left eyelid	CAG ⟶ AZA (75 mg/m^2^ × 7 days)	At least 1	Decreased in size	Died (AML progression)	[11]
8 (2017)	71/F	MDS EB-2	Nasopharynx, tonsil, spleen, multiple lymph nodes, skin	DNR + Ara C ⟶ AZA (100 mg/body × 7 days, as maintenance therapy)	20^*∗*^	Skin: clinical and radiological resolution; others: a marked reduction in the FDG uptake on PET-CT	Alive	[12]
9 (2018)	70/F	Therapy-related MDS (MDS-RS-MLD)	Multiple skin lesions (LC)	A cycle of AZA (75 mg/m^2^) ⟶ 6 cycles of biomodulatory therapy: AZA (75 mg/body × 7 days), pioglitazone and ATRA	7	Clinical resolution	Died (AML progression)	[13]
10 (present case)	89/M	MDS EB-2	Skin	AZA (37.5 mg/m^2^ × 7 days)	16	Clinical resolution	Died (AML progression)	N/A

F: female; M: male; MS: myeloid sarcoma; CMML: chronic myelomonocytic leukemia; MDS: myelodysplastic syndrome; AML: acute myeloid leukemia; EB: excess blasts; AZA: azacitidine; RT: radiotherapy; Ara C: cytarabine; CAG: cytarabine, aclarubicin, and granulocyte colony-stimulating factor (G-CSF); DNR: daunorubicin; FDG: fluorodeoxyglucose; PET-CT: positron emission tomography-computed tomography; N/A: not available; LC: leukemia cutis; MLD: multilineage dysplasia; IDR: idarubicin; allo-HSCT: allogeneic hematopoietic stem-cell transplantation; CLAG: cladribine, high-dose cytarabine, and G-CSF; DLI: donor lymphocyte infusion; FLAG: fludarabine, high-dose cytarabine, and G-CSF; GO: gemtuzumab ozogamicin; 6-MP: 6-mercaptopurine; MDS-RS: MDS with ring sideroblasts; ATRA: all-trans-retinoic acid. ^*∗*^“Ongoing.”

## Data Availability

The data supporting the findings of this study are available from the corresponding author upon reasonable request.
